# “People look at me like I AM the virus”: Fear, stigma, and discrimination during the COVID-19 pandemic

**DOI:** 10.1177/1473325020973333

**Published:** 2021-03

**Authors:** Shinwoo Choi

**Affiliations:** School of Social Work, Texas State University, San Marcos, USA

**Keywords:** Stigma, reflection, race, anti-Asian racism, interminority discrimination

## Abstract

Anti-Asian sentiment is surging because of COVID-19 and there have been incidents of hate crime. This paper presents a reflection by an Asian social work researcher on the impact of this surge on herself and other Asians in America. Reflecting on the history of pandemic, racism against Asian Americans, and anti-Chinese sentiment among other Asian group, the paper provides important points for us to ponder as a community of social workers in the topics of racial justice

*I don’t want to wear a mask in grocery stores because people look at me all weird. They might be thinking I’m already sick, walking around in the public and spreading the virus, you know?*

*It is OK for White people to wear a mask, or to not wear it…. But as Asians, we must be careful.*

*People look at me like I AM the virus. [By wearing a mask] I am being protective [of] my own health and everyone else’s.*

These are the things I am hearing from my Asian friends and acquittances. Because COVID-19 began in China, Asian American communities have been experiencing an increased rate of discrimination and racial microaggressions ranging from subtle hints to physical assaults; even children have been attacked (Loffman, 2020). Inter-minority discrimination has been an element of this surge. I am experiencing intense emotions such as confusion, frustration, exhaustion, and most of all, fear: fear of the unknown, fear of the uncertainty, and fear of possible threat to myself and my family. Friends, relatives, and acquaintances reflect my feelings back to me. Asian Americans are scared and concerned about the virus as much as anyone, and racist attacks add a layer of stress.

People in our society are panicking because of COVID-19 and its economic impacts. I believe that fear from unknowns can transform into other emotions such as anger and hatred. Oftentimes, the hatred is targeted to bigger systems such as governments, media, and other institutions. And people are blaming governments across the world for lack of preparedness and making wrong judgements. However, the accumulated and shared hatred sometimes finds easy targets around us, resulting in racist attacks and intimidation. This essay reflects on fear, stigma, and discrimination during the COVID-19 pandemic from my perspective as a Korean Canadian social work researcher who studies racial microaggressions in the southern part of the United States.

## COVID-19 in historical context

Deadly viruses that originated in animals have created pandemics in humans since the beginning of Neolithic Revolution, approximately 12,000 years ago (Diamond, 1999). Civilizations with dense populations had been particularly vulnerable (Roos, 2020). When a population encounters a “novel” virus, the result can be devasting, such as in the 15^th^ century when smallpox killed more than 90% of the indigenous population of the Americas (Roos, 2020). The flu pandemic that began in 1918 killed almost 50 million people worldwide (Centers for Disease Control and Prevention, CDC, 2018). The exact cause and birthplace of a virus has often been difficult to trace at the time. At first it feels like we have been ambushed out of nowhere; then people may look for someone to blame. The 1918 virus was called “Spanish” flu at the time, but the first known case was in Kansas (CDC, 2018). The first outbreak of H1N1 flu in 2009 was detected in Mexico, but there is no evidence it originated there (Mena et al., 2016). COVID-19 was first detected in China, and but we do not know precisely the cause or where it actually made the transition from animal to human.

There are a lot of speculations about the cause and birthplace of the virus and a lot of looking for someone to blame. The actions (or inactions) that the Chinese government and other governments have taken after the outbreak that worsened it have caused legitimate outrage. However, there is no excuse for directing the resulting anger and hatred to individuals with no real connection to these governments. Yet in doing so those who have committed hate crimes repeat a history in which individuals or groups were punished for or negatively impacted by the relationships between nations or bigger systems. During the World War II, Japanese Americans were discriminated against and forced to relocate to internment camps regardless of their citizenship status (University of California, 2005). The notion that Asian Americans are not part of U.S. society and seen as perpetual foreigners is evident in this and many other incidents in our history. As diplomatic relations between the United States and China worsens in the coming months, the racism ignited by the pandemic seems likely to worsen as well (Liu, 2020).

## Reflections on mask-wearing: Fear, stigma and discrimination

I came to Canada from Korea when I was 14 years old and lived there ten years before coming to the United States as an international student/scholar eleven years ago. I am well-aware of the stereotypes and stigma attached to Asians in North America: as “model minorities,” perpetual foreigners, and outsiders (Sue et al., 2007a). I have devoted my research career to understanding and addressing such stereotypes. Yet I was shocked that wearing a mask might be risky. It was my husband who refused to wear a mask at the supermarket, telling me, “I don’t want to be seen as the Asian who carries virus.” But talking to friends I soon realized that he was not the only one who believed that wearing a mask would put him in more danger than the lowered risk of catching or spreading the virus was worth.

I heard anecdotes about microaggressions from my Asian friends and learned of serious hate crimes across the states which targeted Asian Americans (Margolin, 2020). Microaggressions are “brief and commonplace daily verbal, behavioural, and environmental indignities, whether intentional or unintentional, that communicate hostile, derogatory, or negative racial slights and insults to the target person or group” (Sue et al., 2007b), and, while not as terrifying as hate crimes, they nonetheless cause fear and sadness. The FBI issued a warning in late March, 2020 that some Americans associate the virus with China and that hate crimes against Asians generally might well continue to escalate (Margolin, 2020). An Asian woman was attacked in New York City because she was *not* wearing a mask, while others were attacked because they were wearing a mask. I became afraid of taking even a leisurely walk around our neighbourhood, fearing a hate crime. When I did take a walk with my family, I was hyper-conscious of keeping our distance, seeking to stay well over six feet away if possible, fearing that others would assume we might be infectious because we were Asian. We joked about the stigma, “Being Asians is actually convenient. People just walk away from us. We can keep our social distance easily.”

The experts’ view on the pros and cons of wearing masks has varied as the current pandemic has developed, and in this environment, we feared, as did our friends, that wearing a mask could make us targets. One school of thought argued that wearing a mask does not protect one from infection, and any mask a healthy person, not working in health care, was wearing represented one fewer mask for health care workers and the already sick people (Leung, 2020). The other school of thought was that it is a “common sense” to wear a mask to protect oneself and others, as a nominally healthy person may be asymptomatic or a pre-symptomatic carrier. It is, however, impossible to run a randomized clinical trial to shed light on this question. It is simply unethical and impractical because of time constraints in the current crisis. In the absence of better information, some people say that it’s better to wear a mask. On April 3 the CDC finally began to recommend wearing masks in public (CDC, 2020). I was enormously relieved, as I felt we could wear a mask without fear of stigma.

Once liberated from the dilemma, I realized I had been thinking ill of other Asians who wore masks before the CDC guidelines changed. I had thought, “Wow, look at all the Asians wearing masks. Other people will see them as Chinese carrying the virus.” I was being conscious of how other Asians were being perceived by non-Asians out of fear that we might experience discrimination because they stoked non-Asians’ anger against us. When I saw White, Black or Latinx people in masks and medical grade gloves, I perceived them as cautious and smart. When I saw White people without them, I found myself giving them credit for not being overly panicky and keep being calm. In reflecting on these feelings, I could see that I have accumulated the unconsciously shared beliefs that White people are “good” “fair-minded” and “descent” (Sue et al., 2007a, 2007b).

I never tried to protect myself from discrimination by asserting that I am not Chinese, but I understood the impulse. Hate crimes against Asian made non-Chinese eager to assert their differences. Friends joked about wanting a tee-shirt saying, “I am from Korea, NOT from China” or “Made in Vietnam, NOT CHINA” and in fact multiple online sites sell such products (Amazon, n.d.). My Asian friends from various ethnic groups—Korean, Taiwanese, and Japanese—expressed hatred towards China. When I told a Korean friend that we should not blame the Chinese people for actions their government might have taken, she said, “Government or people, they are the same!” I found this quite alarming. I wanted to ask, “So, is it okay if Korean government did something wrong and we experience discrimination?” But I understand that there are cultural explanations for this logic. First, since collectivism, in which an individual is often seen as a part of the bigger group that they belong to, is part of most Asian cultures (Robson, 2017), distinguishing the individuals from wrongdoings of their government is a novel concept. In my non-Chinese friends’ minds, all they want to express is their “non-Chinese” ethnicity. Second, unfortunately, it is common for minority groups to discriminate against each other when they are being marginalized and resources are scarce (Burson and Godfrey, 2018). My friends’ impulses echo with history. Many Asian American groups suffered discrimination after Pearl Harbor in 1941 (Chan, 2019). Those who were not Japanese sought protection in that fact, and some supported anti-Japanese efforts, either explicitly or implicitly. A history of Japanese imperialism in Asia made it easy for people to draw on their own existing resentment. But it is also true that some showed support towards Japanese Americans and there were solidarity efforts through public speeches and writings (Chan, 2019). Today, scholars and activists are encouraging Asian Americans to turn to solidarity among Asian ethnic groups and other minority groups in recognition that we are stronger together (Chou, 2020).

Indeed, the mask question is a hard one for other people of color. I was surprised to learn that Black men are afraid to wear a mask in public. Surely no one, I reasoned, thought they had a particular propensity for the virus? But I learned that they were concerned that a mask might be associated with criminality, in line with persistent stereotyping of Black men as violent and lawless (Sue et al., 2007a, 2007b).

Learning about how different marginalized groups are experiencing discrimination, on top of the fearing for virus, was extremely fatiguing. The virus itself is scary enough. If people cannot wear a mask without added fear, we are living in a dystopia.

## Last reflections as the pandemic continues

Virtually no one alive today remembers a pandemic as serious as the one we now face. The future of the virus and its impact on mankind is still unknown. It feels surreal and bizarre. I dream of my family using our unused admissions tickets to Disney World or going to a Maroon 5’s concert. When I wake up, I feel sad again. Nobody knows how we will go back to being “normal” again.

This is a surreal time of many tragic events. Already vulnerable families are experiencing financial crises as they lose their jobs, their childcare, and their health care without preparation or support. Thousands of people are quarantining with abusers and death tolls are mounting. In times of uncertainty and fear hatred can grow. Then the consequences can be devastating. As a social work researcher who studies racial discrimination, observing the intensified anti-Asian discrimination, from non-Asians and Asians during pandemic fuels my research interests. But as a member of the society and humanity, it is deeply troubling and disheartening.

Prominent historian Yuval Harari called on the countries of the world to work together to solve the biggest challenge in human history in the Financial Times’ article (2020). In today’s world, every society is interconnected closely. The pandemic has exposed this. We are scared and angry. I desperately want the answers, too. When will a vaccine and cure be available? What caused the emergence of this deadly virus? Just like many people, I want someone to take responsibility for the pain that the world is going through. But we cannot allow the fear of unknown to target the innocent and equally scared. Although it is difficult, we should support and show compassion to each other in this time.
